# Using Machine Learning to Evaluate the Value of Genetic Liabilities in the Classification of Hypertension within the UK Biobank

**DOI:** 10.3390/jcm13102955

**Published:** 2024-05-17

**Authors:** Gideon MacCarthy, Raha Pazoki

**Affiliations:** 1 Cardiovascular and Metabolic Research Group, Division of Biomedical Sciences, Department of Life Sciences, College of Health, Medicine and Life Sciences, Brunel University London, London UB8 3PH, UK; 2 MRC Centre for Environment and Health, Department of Epidemiology and Biostatistics, School of Public Health, St Mary’s Campus, Norfolk Place, Imperial College London, London W2 1PG, UK

**Keywords:** the receiver operation characteristic (ROC), area under the curve (AUC)

## Abstract

**Background and Objective:** Hypertension increases the risk of cardiovascular diseases (CVD) such as stroke, heart attack, heart failure, and kidney disease, contributing to global disease burden and premature mortality. Previous studies have utilized statistical and machine learning techniques to develop hypertension prediction models. Only a few have included genetic liabilities and evaluated their predictive values. This study aimed to develop an effective hypertension classification model and investigate the potential influence of genetic liability for multiple risk factors linked to CVD on hypertension risk using the random forest and the neural network. **Materials and Methods:** The study involved 244,718 European participants, who were divided into training and testing sets. Genetic liabilities were constructed using genetic variants associated with CVD risk factors obtained from genome-wide association studies (GWAS). Various combinations of machine learning models before and after feature selection were tested to develop the best classification model. The models were evaluated using area under the curve (AUC), calibration, and net reclassification improvement in the testing set. **Results:** The models without genetic liabilities achieved AUCs of 0.70 and 0.72 using the random forest and the neural network methods, respectively. Adding genetic liabilities improved the AUC for the random forest but not for the neural network. The best classification model was achieved when feature selection and classification were performed using random forest (AUC = 0.71, Spiegelhalter z score = 0.10, *p*-value = 0.92, calibration slope = 0.99). This model included genetic liabilities for total cholesterol and low-density lipoprotein (LDL). **Conclusions:** The study highlighted that incorporating genetic liabilities for lipids in a machine learning model may provide incremental value for hypertension classification beyond baseline characteristics.

## 1. Introduction

Approximately 1.28 billion people aged 30 to 79 have hypertension worldwide [1], and it continues to rise globally, causing a significant socioeconomic burden due to low awareness and poor control [2]. Hypertension significantly increases the risk of cardiovascular diseases (CVD), including stroke, heart attack, heart failure, and kidney disease, contributing to the global disease burden and premature mortality [1,3,4].

Every year the burden of hypertension and related CVD is increasing in the United Kingdom (UK). As of 2017, hypertension prevalence in England was estimated at around 26.2% among adults [5]. It is responsible for more than half of all strokes and heart attacks, costing the National Health Service (NHS) more than £2.1 billion per year [6].

The current guidelines [7,8,9] suggest lifestyle modification and the use of blood pressure-lowering medication to prevent hypertension and its consequences. Medication is often successful in lowering blood pressure and reducing the risk of hypertension-related CVD and stroke. Lifestyle modifications also offer benefits including reduced drug costs, improved control of other comorbidities such as diabetes and hypercholesterolemia, and avoiding preventable pharmacological therapy [10]. The current guidelines have remained silent on the genetic components of hypertension, which are quantifiable at birth and may be used to determine an individual’s lifelong disease risk before clinical risk factors are established [11], allowing adequate time to determine lifetime measures to lower hypertension risk, particularly in a high-risk group.

Genome-wide association studies (GWAS) have identified numerous multiple single nucleotide polymorphisms (SNPs) associated with hypertension and/or high blood pressure levels [12,13,14,15,16,17]. Developing methods to incorporate genetic factors into classification models of hypertension has the potential to improve hypertension classification, management, and control.

Previous studies have used standard statistical techniques or machine learning to predict hypertension [18,19,20,21,22,23,24,25,26,27,28,29]. However, most of these studies only focused on non-genetic risk factors to predict hypertension [18,19,20,21,22,23]. The studies that included genetic risk factors only focused on single SNPs at a time [26,30], or gene expression [27], or a single genetic risk score [25]. Our study offers the incorporation of multiple genetic liabilities into machine learning methods above and beyond previous studies. An example of previous studies includes a recent study in rural Chinese populations [25] that incorporated a single hypertension polygenic risk score (PRS) and showed improvement in incident hypertension prediction using several machine learning techniques.

Several studies [31,32,33] have used a method called multi-polygenic score or meta genetic risk score (metaGRS) that combines several PRSs into regression models for complex diseases, including CVD, and have shown that including multiple genetic factors improves the prediction model’s accuracy compared to using one genetic liability. However, these [31,32,33] did not incorporate the metaGRS within machine learning methods and did not consider hypertension as the outcome in their models. The conventional statistical techniques utilized in previous studies encounter challenges in identifying complex, nonlinear relationships within datasets and exhibit a limited ability to generalize to unseen data (test data). Their constraints emphasize the need for supplementary methodologies, such as machine learning techniques, that offer greater flexibility and robustness in handling complex data structures and achieving accurate predictions. Furthermore, previous studies have not considered the inclusion of genetic liabilities for multiple CVD risk factors to predict hypertension. It is yet to be determined whether machine learning techniques could be applied to enhance hypertension prediction models derived from multiple genetic liabilities for CVD risk factors.

Recent studies have provided evidence for genetic correlations between hypertension and type 2 diabetes [34], adiposity traits [35], lipids traits [36,37], and smoking traits [38]. In the current study, we created genetic liabilities using these risk factors and used machine learning models to evaluate the best combination of genetic liabilities and clinical factors that could optimize the classification of hypertension in the European ancestry population.

## 2. Material and Method

### 2.1. Ethical Approval

The UK Biobank (UKB) received ethical approval from the Northwest Multi-centre Research Ethics Committee as a Research Tissue Bank approval, and all the participants provided informed consent. This study is performed using the UKB data under application number 60549. Additionally, we obtained ethics approval from Brunel University London, College of Medicine, and the Life Sciences Research Ethics Committee to work with secondary data from the UKB (reference 27684-LR-Jan/2021-29901-1).

### 2.2. Study Population

UKB is a prospective observational study with more than half a million participants aged between 40 and 69 years. The participants were recruited between 2006 and 2010 across 22 centres located throughout the United Kingdom (UK). The full description of the UKB study as well as the data collected and a summary of the characteristics are publicly available on the UKB website (www.biobank.ac.uk, accessed on 20 June 2021) and elsewhere by Sudlow and colleagues [39]. In brief, during the recruitment, detailed information about socio-demographics, health status, physician-diagnosed medical conditions, family history, and lifestyle factors was collected via questionnaires and interviews. Several physical measurements, including height, weight, body mass index (BMI), waist–hip ratio (WHR), systolic blood pressure (SBP), and diastolic blood pressure (DBP), were obtained. The records of participants in the UKB project were accordingly linked to Hospital Episode Statistics (HES) data, as well as national death and cancer registries.

**Figure 1:**
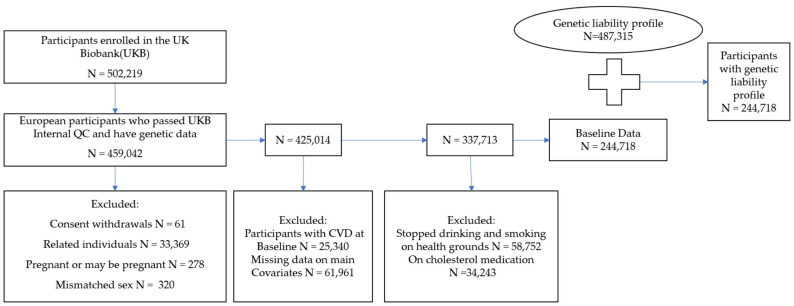
**The flowchart of the study participant selection**. UK Biobank (UKB) data had 502,219 participants at the beginning of this study. We extracted 459,042 participants of European ancestry who have passed UKB internal quality control (QC) and have genetic data. The final dataset included 244,718 participants who met the inclusion criteria for whom a genetic liability profile was generated.

The current study is based on a subset of unrelated individuals of European ancestry (n = 244,718; Figure 1). In brief, we used 40 genetic principal components created centrally by the UKB and applied the k-means clustering method on 502,219 UKB participants to identify individuals of European descent. We then obtained genetic data from the individuals who had passed the UKB internal quality control and had genotype data (n = 459,042). We excluded individuals (n = 25,340) who had been diagnosed with a stroke, heart attack, or angina before or at baseline. This strategy helps to adjust for pre-existing CVDs and minimizes the possibility of confounding. We excluded participants who had withdrawn their consent (n = 61), pregnant individuals, or those uncertain about their pregnancy status (n = 278). We additionally excluded individuals with mismatched genetics and self-reported (n = 320) to avoid potential inconsistencies in data reporting. Using the kinship cut of 0.0884 for third-degree relatives, we further excluded participants who were up to second-degree related (n = 33,369). Furthermore, individuals who were on cholesterol-lowering medication (n = 34,243), stopped smoking or drinking due to health reasons or doctor’s advice (n = 58,752), and participants with missing data on the potential confounders (n = 61,961) were excluded from the dataset, leaving a final 244,718 unrelated individuals of European ancestry for our analyses.

### 2.3. Genotyping and Imputation

The UKB conducted all the DNA extraction, genotyping, and imputation. The detailed processes have been discussed elsewhere [40,41,42]. In brief, blood samples from participants were obtained at UKB assessment centres, and DNA was extracted and genotyped using the UKB Axiom Array. The genotype imputation was conducted by UKB using the IMPUTE4 tool. Three reference panels—Haplotype Reference Consortium, UK10K, and 1000 Genomes phase 3—were used for the imputation. The genetic principal components and kinship coefficients were calculated centrally by UKB to account for population stratification and identify related individuals [40,42].

### 2.4. Definition of the Outcome

Our main outcome is hypertension, which was defined as (1) the presence of a recorded SBP ≥ 140 mmHg or a DBP ≥ 90, or (2) hypertension diagnosed by a doctor, or (3) a record of using blood pressure-lowering medication at baseline [43]. In the UKB, two blood pressure readings were obtained a few minutes apart using a standard automated device or manual sphygmomanometer (www.ukbiobank.ac.uk, accessed 20 June 2021). We calculated both mean SBP and mean DBP from two automated or two manual readings of blood pressure measurements. For participants with one manual and one automated blood pressure reading, the average of these two values was used. For individuals with a single blood pressure measurement (one manual or one automated blood pressure reading), the single measurement was used for approximating the participant’s blood pressure value. For the participants who self-reported to be taking blood pressure-lowering medication, we added 15 mmHg to SBP and 10 mmHg to DBP [44]. The participants with missing blood pressure readings were excluded.

### 2.5. Demographics and Clinical and Lifestyle Features

In the statistical analysis, factors such as age, sex, BMI, diabetes mellitus, total cholesterol (TC), low-density lipoprotein (LDL), high-density lipoprotein (HDL), smoking status, drinking status, and sedentary lifestyle were included. Diabetes was defined as a record of diabetes diagnosed by a doctor, or using insulin medication, or a record of serum level of haemoglobin A1c (HbA1c) ≥ 48 mmol/mol (6.5%), or glucose level ≥ 7.0 mmol/dL [45]. Smoking and alcohol consumption data were collected through a self-reported questionnaire by the UKB and were classified into current, previous, and never.

We calculated a sedentary lifestyle variable by approximating the total self-reported hours per day the participants spent on (1) driving, (2) using a computer, and (3) watching television. We considered 30 min of sedentary behavior if individuals indicated that they spent less than an hour per day driving, or watching television, or using a computer. In this study, the demographics and clinical and lifestyle (non-genetic) features were selected as conventional risk factors for CVD that have been used often in previously published work of ours and others [28,44].

### 2.6. Computation of Genetic Liabilities

#### SNP Selection

We selected a list of genetic variants in the form of SNPs (Table 1 and Supplementary Data S1–S10) that were previously identified as associated with ten CVD risk factors, including type 2 diabetes [46], two adiposity traits [47,48], three smoking traits [49], and four lipid traits [50] at a GWAS significant threshold (*p*-value < 5.0 × 10^−8^) in the European population. Linkage disequilibrium (LD) measures the non-random linkage of alleles at different loci on the same chromosome in a population. SNPs are said to be in LD when the frequency of association between their alleles exceeds what would be expected from a random assortment [51]. LD between two loci is determined statistically using metrics like r^2^. This metric measures the level of connection between alleles at the two loci. The SNPs used in calculating the genetic liabilities were pruned with the LD pruning procedure employed in SNPclip incorporated within the LDlink online tool (https://ldlink.nih.gov/?tab=home, accessed on 20 July 2021). A minor allele frequency (MAF) of 0.01 and r^2^ threshold of 0.1 was used in LD pruning. Duplicate SNPs, not biallelic SNPs, SNPs with MAF less than 0.01, and SNPs in LD with other SNPs (r^2^ > 0.1) were excluded. We used the final list of selected LD-pruned SNPs to estimate genetic liabilities for all the ten traits in the current study using PLINK version 1.9 [52]. To allocate weight to each SNP, we used the effect sizes estimated for the association of the SNPs with each of the traits mentioned in Table 1. The effect sizes (Supplementary Data S1–S10) were obtained from previously published, publicly available GWAS summary statistics data provided for these SNPs within the GWAS Catalog website (https://www.ebi.ac.uk/gwas/, accessed on 12 July 2021). PLINK uses a weighted method, where the effect size (beta coefficient) of each SNP is considered as weight and is multiplied by the number of risk alleles an individual carries. The product is then summed across all SNPs to produce genetic liability for each person. We standardized all the genetic liabilities (mean-centred with standard deviation 1).

### 2.7. Statistical Analysis

We summarized the categorical variables using frequencies and percentages, and the continuous variables were expressed as the mean (SD). When comparing the characteristics differences between the hypertensive and non-hypertensive groups, the nonparametric test (Wilcoxon rank sum test) was utilized for continuous variables as the assumptions for the parametric *t-test* may not be met. The chi-squared test was applied to compare the hypertensive and non-hypertensive groups for categorical variables. We used univariable logistic regression to determine the strength and direction of the relationship between individual features with hypertension without considering other variables. We also used multivariable logistic regression to assess the independent effects or association of each feature while controlling for the effects of other features in the model. The statistical significance of the association was defined, where the associations demonstrated a 2-sided *p*-value less than 0.05 (see Supplementary Tables S1 and S2).

### 2.8. Data Preprocessing and Splitting

We excluded participants who had missing values for essential variables. Features included in our machine learning algorithm are presented in Table 2.

All categorical variables were labelled, including gender (0 = female, 1 = male), smoking status (0 = never, 1 = previous, 2 = current), alcohol consumption status (0 = never, 1 = previous, 2 = current), diabetes (0 = no, 1 = yes), and our outcome variable, hypertension (0 = no, 1 = yes). Our numerical variables were all measured on different scales. To ensure that all the numerical variables contribute equally to our model [53], we scaled them to a given range, using a “min–max” approach.

**Figure 2:**
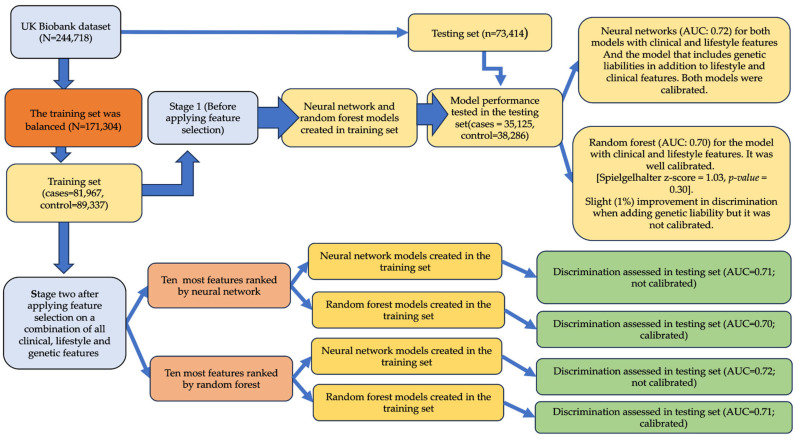
**Overview of the study and the construction of machine learning models:** The flowchart of the study design. The data were split into training and testing sets. Stage one models were built in the training set without selection, and their performances were assessed in the testing set. Stage two models were built after the feature selection technique was applied, and their performances were evaluated in the testing set.

In machine learning, data splitting is a common practice used for evaluating the performance of a prediction model. This involved splitting the available dataset into training and testing sets. The training set is for training the machine learning model, and the testing is used to assess the model’s performance. In this study, we employed the train-test split approach [54] to randomly partition the dataset at a ratio of 70:30 (Figure 2) into a training set (70%; n = 171,304; case = 81,967 and control = 89,337) and a testing set (30%; n = 73,414) using the “*createDataPartition*” function in the R-package version 4.2.2. This approach ensures both our training set and testing set capture the underlying distribution of the data.

The models were trained in the training set, and the performance of the models in terms of discrimination ability (defined as the model’s capacity to distinguish between persons with and without outcomes) was assessed in the testing set (n = 73,414; Figure 2). To this end, we constructed the receiver operating characteristic curve (ROC) for each model and calculated the area under the curve (AUC) with 95% confidence intervals (CIs) [55,56,57]. The AUC ranges from 0.5 to 1.0, with 0.5 indicating no better discrimination than chance and 1.0 representing perfect discrimination power.

### 2.9. Handling Data Imbalance

Models trained on imbalanced datasets may become biased towards the dominant class, predicting the minority class incorrectly [58]. A binary classifier, which is the case in the current study, trained on a balanced dataset typically outperforms a model trained on an imbalanced dataset [59]. An imbalanced training dataset may lead to overfitting the majority class due to their higher prior probability [60]. This means that the minority class may be misclassified more frequently as compared with the majority class. This issue could lead to incorrect prediction and that some model performance metrics, such as accuracy, may be distorting the conclusions [60]. Balancing the training set is an approach that could prevent overfitting, reduce bias, and ensure that the model learns to successfully classify groups in addition to improving the accuracy of prediction on the testing data. To balance the number of events in the training set before training the models, we utilized the random over-sampling using a bootstrapping method implemented within the “*ROSE*” (Random Over-Sampling Examples) package [61]. To deal with imbalanced data and balance the class distribution in the dataset, the ROSE package generates synthetic samples for the minority class [61].

### 2.10. Machine Learning Model Construction

In comparison with traditional statistical techniques, machine learning algorithms are flexible and free of prior assumptions (e.g., the type of error distribution) and can capture the complicated, nonlinear relationships between predictors. These algorithms automate decision-making processes using models that have been trained on historical data [62]. They can analyse various data types and integrate them into predictions for disease risk [63]. Machine learning algorithms with and without genetic data were used in the prediction of hypertension in the European population, including support vector machines, decision trees, random forest, neural network, and extreme gradient boosting (XGBoost) [64]. For this study, we considered two machine learning-based classifiers, the random forest and the neural network, which have been shown in many studies to have been most promising in the classification of hypertension among individuals of European ancestry [64].

The random forest is a powerful machine learning algorithm that constructs an ensemble or forest of decision trees that are often trained using the bagging method. Each decision tree is constructed on a random subset of the training set and a random subset of the features. This keeps the trees from becoming overly correlated and, hence, overfitting the data [65]. Using the training set, the random forest models were constructed with the “*ranger*” package in the R-programme [66] with hyperparameters set to 500 trees and 10 nodes. The optimal model was selected based on an out-of-bag (OOB) estimate of the error rates in the training set. In a random forest model, the maximum number of features that can be considered for splitting at each node of the decision trees within the ensemble was determined and reported using the “*mtry*” parameter within the “*ranger*” package (Supplementary Table S1).

The neural network is another powerful machine learning algorithm that automatically learns from patterns between the inputs and the output within the data [67]. The neural network consists of interconnected processing nodes organized in three layers: input, hidden, and output layers (Supplementary Figure S1). The input layer is connected to the hidden layer with updated weight, which is then connected to the output layer [68]. In the construction of our neural network models, the optimal number of hidden layers was identified as 5 hidden layers. The neural network classifiers were constructed with the “nnet” function [69] using 5-fold cross-validation implemented in the R-programme “caret” package. We used the following hyperparameters: The regularization parameter used to prevent overfitting in the neural network by penalizing large weights (decay) was set to 0. Setting it to 0 means that no weight decay is applied. The maximum number of iterations (epochs) for training the neural network (maxit) was set to 100, and the maximum number of weights in the neural network (maxNWTs) was set to 1000.

Overfitting and underfitting are two frequent machine learning issues that can have a significant influence on model performance and generalizability [70]. Overfitting occurs when a model fits the training set but performs poorly on the testing or an unknown dataset, resulting in low training error but high test error. To minimize overfitting within the neural network models and to produce more reliable estimations of the models’ prediction abilities on the testing set [71,72], we performed a 5-fold cross-validation on the balanced training set (n = 171,304). The ROC [73] was used to select the optimal model (based on the largest ROC value estimated at 0.70; see Supplementary Table S2). This implies that models with ROC values greater than 0.70 in the testing set would improve prediction.

We adopted a two-stage approach in the construction of the machine learning algorithm.

#### 2.10.1. Stage One Models

In stage one, we built models without feature selection in the training set (n = 171,304). In the first subset of models, we used the random forest method [65,66], and in the second subset of models, we used the neural network (Figure 2). For each of these methods, we used two different sets of features: (1) conventional risk factors that included baseline characteristics (age, sex, BMI, diabetes mellitus, smoking status, drinking status, TC, HDL, LDL, and sedentary lifestyle; and (2) full set of features included all the baseline characteristics above together with additional genetic variables, including ten genetic liabilities.

The optimal number of features used for splitting at each of the decision trees was identified as three features in the construction of the random forest model with ten conventional risk factors vs. four features in the random forest model with the additional ten genetic liabilities. Both random forest models above showed a prediction error of 0.22.

In the testing set, we evaluated and compared the performance of (1) the random forest model with and without genetic liabilities and (2) the neural network model with and without genetic liabilities. The performance of our machine learning models was evaluated using the AUC, accuracy, sensitivity (recall), and F1 score.

#### 2.10.2. Stage Two Models

In stage two, to improve model performance, we utilized a feature selection strategy to select the most relevant features, eliminate unnecessary noise or random fluctuation in the data, and prevent the problem of overfitting (induced by the presence of irrelevant features). Both the random forest and neural network approaches were used as the feature selection method. The most important features were ranked based on their importance score and illustrated using variable importance plot (*vip*) function within the *caret* package. Regardless of the method used in the feature selection step, we further used the top ten most important features identified to further develop classification models using random forest and neural network. This approach created four different analysis paths to hypertension classification including the path (1) where the feature selection model was random forest, and the classifying method was random forest as well; path (2) where random forest was used as the feature selection method, and the classification method was neural network; path (3) where the feature selection model was neural network, and the classification method was neural network as well; path (4) where neural network was the feature selection method, and the classification method was random forest (see Figure 2). In the testing set, we used the AUC (see above) to assess the performance of these four models built with the ten most important features selected. Stage one and stage two resulted in the construction and testing of a total of eight models.

### 2.11. Model Performance Assessment Using Calibration

We used a calibration curve and Spiegelhalter z score test to examine the models’ calibration [74,75]. Model calibration measures the ability of a model to accurately predict an outcome [76,77]. In the calibration curve, the *Y*-axis represents the observed probability, and the *X*-axis represents the predicted probability of developing a disease. The calibration curve includes a diagonal line (the ideal line), which is the prediction of the ideal model. A model is said to be well-calibrated if the calibration curve stays close to the line of perfect calibration (45 degrees with an intercept of 0 and a slope of 1). Overestimation and underestimation are identified by a curve below and above the ideal calibration line, respectively. The Spiegelhalter z test is a statistical test used to assess the calibration accuracy of a risk prediction model. A perfectly calibrated model (i.e., when the predicted probabilities match the observed values) has a Spiegelhalter z score of zero, while a value close to zero indicates good calibration, and a value far from zero indicates poor calibration. A positive Spiegelhalter z score indicates that the model is over-calibrated (i.e., the predicted probability of the outcome is too high), while a negative Spiegelhalter z score indicates that the model is under-calibrated (i.e., the predicted probability of the outcome is too low).

To confirm the overall accuracy of the models, we also calculated the Brier score [78], which is the mean square error (MSE) between observed and predicted outcomes. The Brier score evaluates both the calibration and discrimination ability of a model [77]. The scores range from 0 to 1, with lower scores suggesting superior calibration. Brier scores approaching 0 imply that the model has been adequately calibrated and discriminated. We used the validation probability (*val.prob*) function from the “*rms*” package in the R-programme to generate calibration curves, Spiegelhalter z test, and Brier score.

### 2.12. Net Reclassification Index and Integrated Discrimination Index

We assessed the performance of well-calibrated models using the net reclassification index and integrated discrimination index statistics. The net reclassification improvement is a commonly used metric to compare the relative ability of two models to classify individuals as low- and high-risk [79]. A positive net reclassification index value indicates that the new model correctly reclassifies more individuals into higher- or lower-risk categories compared to the old model. Conversely, a negative net reclassification index value suggests that the old model is better at reclassifying individuals than the new model.

The integrated discrimination index statistic is used to measure the improvement in the ability of two models to distinguish between event and non-event [80,81]. A positive integrated discrimination index value implies an improvement in the model’s discriminative ability, while a negative integrated discrimination index value suggests a deterioration in the discriminative ability of the new model. In this study, we used the “*reclassification*” function from the “*PredictABEL*” packages in the R-programme to obtain the net reclassification index and integrated discrimination index values. The discrimination ability, calibration, and reclassification results are depicted further in Figure 2. All the analysis was performed with R-program (www.r-project.org; access data December 2022) version 4.2.2. For reproducibility, we set the seed of the random number generator to a value of 500 throughout this analysis. The code for the analyses has been generated and accessible to the public through Github links below:

1. https://github.com/GMaccarthy/NN_with_Imbalanced_Trainingset, accessed on 30 April 2024;
2. https://github.com/GMaccarthy/NN_with_Balanced_Trainingset, accessed on 30 April 2024;
3. https://github.com/GMaccarthy/RF_Balanced_trainingset, accessed on 30 April 2024;
4. https://github.com/GMaccarthy/RF_Imbalaced_Trainingset, accessed on 30 April 2024.

## 3. Results

### 3.1. Baseline Characteristics of the Participants

A total of 244,718 unrelated individuals of European ancestry from the UKB were included in this study (Table 3). The average age of the participants was 55.4 ± 7.98 years old, and 141,931 (58.0%) participants were female. The sample contained 7011 (2.9%) participants with diabetes. The majority (n = 229,539; 93.8%) of the participants reported to be current alcohol drinkers, and 164,847 (67.4%) reported to have never smoked. The average BMI was 26.8 (4.58) kg/m^2^. The sample included 117,095 (47.8%) participants with hypertension. There were statistically significant differences in all baseline characteristics between the hypertensive and non-hypertensive groups (Table 3). More women were hypertensive than men (52.4% vs. 47.6%; *p*-value < 0.001). The hypertensive participants were older (57.6 ± 7.53 vs. 53.4 ± 7.84 years; *p*-value < 0.001), had higher BMI (28.0 ± 4.83 kg/m^2^ vs. 25.8 ± 4.06 kg/m^2^; *p*-value < 0.001), had higher TC levels (6.05 ±1.06 vs. 5.79 ± 1.04 mmol/L; *p*-value < 0.001), and spent more hours per day having sedentary lifestyle (4.87 ± 2.39 vs. 4.50 ± 2.33 h per day; *p*-value < 0.001) than the non-hypertensive participants.

All the demographic, clinical, and lifestyle features included in the study had a statistically significant association with hypertension (Table 3, Supplementary Tables S3 and S4).

### 3.2. Stage One Models

**Figure 3:**
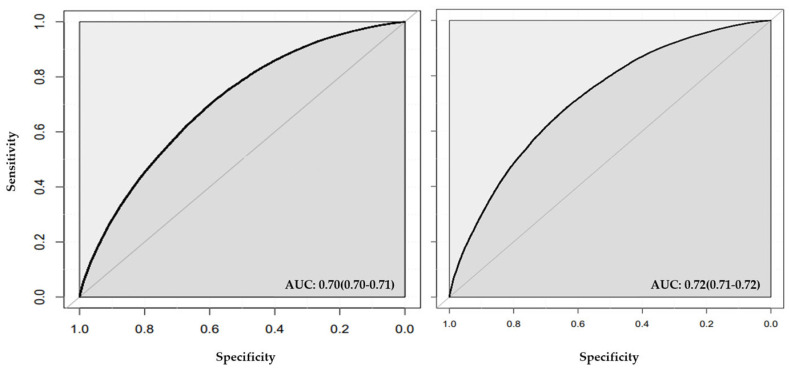
**ROC plot for models with conventional risk factors in stage one.** The figure shows the area under the curve (AUC) for both random forest (left panel) and neural network (right panel).

In stage one (Figure 2), the models incorporating conventional CVD risk factors (i.e., age, sex, BMI, diabetes, smoking status, drinking status, TC, HDL, LDL, and sedentary lifestyle) achieved AUCs of 0.70 (95% CI = 0.70, 0.71; Table 4 and Figure 3), accuracy of 0.65 (95% CI = 0.65, 0.66), a sensitivity of 0.68, and F1-Score of 0.64 using the random forest method (Table 4). The calibrations measured by Spiegelhalter’s z score were 1.03 (*p*-value = 0.30, calibration slope = 0.98) for random forest (Table 4 and Supplementary Figure S2). We observed AUC of 0.72 (95% CI = 0.71, 0.72), accuracy of 0.66 (95% CI = 0.65, 0.66), a sensitivity of 0.69, and F1-Score of 0.66 using neural network (Table 4 and Figure 3). Spiegelhalter’s z score was estimated as −14.39 (*p*-value = 6.4 × 10^−47^, calibration slope = 1.18) using neural network (Table 4, Figure S3).

**Figure 4:**
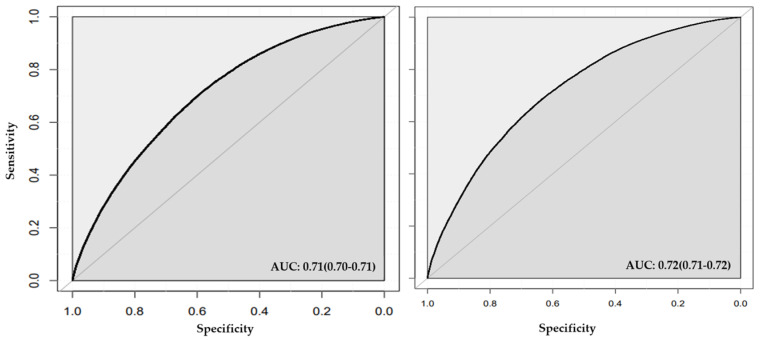
**ROC plot for stage one models including conventional risk factors and genetic liabilities.** The figure shows the AUC for random forest (left panel) and neural network (right panel).

The addition of genetic liabilities resulted in a slight improvement in the AUC only in random forest model (AUC = 0.71; Table 4 and Figure 4). We observed a Spiegelhalter’s z score of −5.64 (*p*-value = 1.7 × 10^−8^, calibration slope = 1.06; Table 4 and Supplementary Figure S2). A Spiegelhalter’s z score of −14.44 (*p*-value = 3.0 × 10^−47^, calibration slope = 1.18) was observed for neural network models.

### 3.3. Stage Two Models

**Figure 5:**
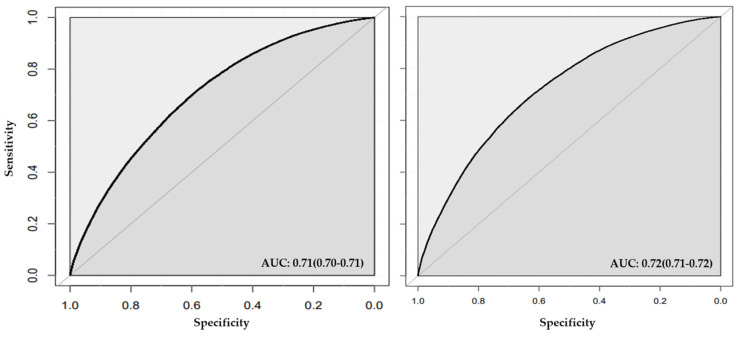
**ROC plot for stage two models created with features selected by random forest that included conventional risk factors and genetic liabilities.** Area under the curve (AUC) is illustrated for classification by random forest (left panel) and neural network (right panel).

**Figure 6:**
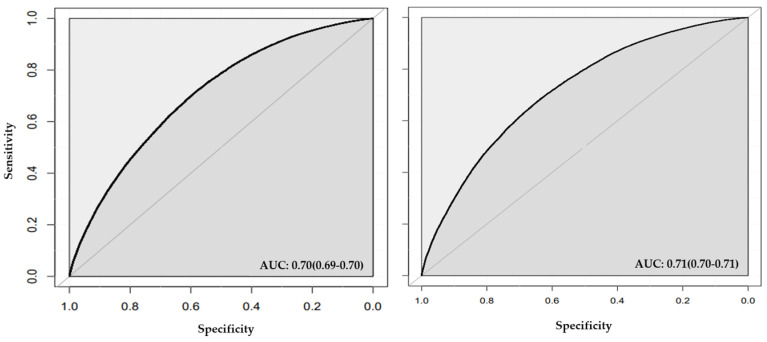
**ROC plot for stage two models created with features selected by neural network that included conventional risk factors and genetic liabilities.** Area under the curve (AUC) is illustrated for classification by random forest (left panel) and neural network (right panel).

In stage two (Figure 5 and Figure 6), random forest feature selection identified feature age as the most important classifying feature for hypertension, followed by sex, BMI, TC, LDL, sedentary lifestyle, HDL, TC genetic liability, LDL genetic liability, and smoking status. (Supplementary Figure S4). Feature selection using neural network identified HDL as the most important feature, followed by TC, LDL, sedentary lifestyle, LDL genetic liability, BMI, TC genetic liability, age, WHR genetic liability, and HDL genetic liability (Supplementary Figure S5).

The model in stage two that was well-calibrated and achieved an improved AUC among the four models developed in this stage was the model built with the important features (Supplementary Figure S4) selected and classified using random forest (see Methods). The model achieved an AUC of 0.71 (95% Cl = 0.70, 0.71) and a Spiegelhalter’s z score of 0.10 (*p*-value = 0.92, calibration slope = 0.99; Supplementary Figure S6). The model showed accuracy of 0.65 (95% CI = 0.64, 0.65), sensitivity of 0.66, and F1-Score of 0.64 (Table 4 and Figure 5).

### 3.4. Reclassification Index Analysis

Three models with a random forest classifier, including one from stage one and two from stage two analysis, were identified as well-calibrated. These models were included in the reclassification index analysis where the model from stage one was used as the reference (that is the model which included all conventional CVD risk factors and used random forest as classifying method; Figure 2).

The stage two model which was built using random forest as both feature selection and classification method (Table 5) showed a slightly improved reclassification compared with the reference model indicated by a net reclassification index of 0.06 (95% CI = 0.05, 0.08; Table 5). This model showed an integrated discrimination index of 1.7 × 10^−3^ (95% CI = 9.0 × 10^−4^, 2.5 × 10^−3^; Table 5).

The stage two model which was built using neural network as feature selection method and random forest as classification method showed a deteriorated reclassification compared to the reference model, indicated by a net reclassification index of −010 (95% CI = 0.12, 0.09; Table 5). This model showed an integrated discrimination index of −0.01 (95% CI = −9.3 × 10^−4^, −0.01; Table 5).

## 4. Discussion

This is the first large-scale research that has been conducted on hypertension classification using machine learning that investigates the prediction value of a combination of genetic liabilities for type 2 diabetes, adiposity traits, lipid traits, and smoking traits in a single model. Aided by machine learning, we used a European dataset with 244,718 participants from the UKB and identified the best integrated predictive models for the classification of hypertension. We found that incorporating multiple genetic risk factors into prediction models could lead to a minor but statistically significant improvement in the classification ability and reclassification of the models beyond conventional risk factors. Of all the genetic liabilities we considered, those estimated for TC and LDL cholesterol were identified to be a combination that could improve the classification of hypertension compared with the model without any genetic factors. This is the first study that identifies the predictive value of the genetic liability of lipid traits in the hypertension classification. Several cohort studies have found a link between high cholesterol levels [82,83] as well as dyslipidaemia [84,85] and an increased risk of developing hypertension. Dyslipidaemia is known to impair the functional and structural features of the arteries and cause atherosclerosis [86]. These changes may compromise blood pressure control, predisposing individuals with dyslipidaemia to hypertension.

Previous literature has only described traditional statistical techniques and machine learning models to predict/classify hypertension, mainly using non-genetic risk factors [18,19,20,21,22,23,24]. The studies that included genetic risk factors only used single SNPs at a time [26,30] or gene expression [27]. Furthermore, a recent study [25] that used machine learning models and incorporated genetic liability only examined one genetic liability at a time. Niu and colleagues [25] utilized three machine learning models, including random forest and neural network, which incorporated a genetic liability component to predict hypertension in rural China. The models included an Asian ancestry hypertension PRS derived from 13 SNPs. The authors observed that integrating hypertension PRS into models improved hypertension incidence prediction and risk classification (AUC random forest = 0.84; AUC neural network = 0.80). Vaura and colleagues [29] incorporated PRS for SBP and DBP in Cox models to assess predictive values of these genetic markers in the risk of incident hypertension prediction using Cox proportional hazards models. The authors observed that including PRS for blood pressure in the clinical prediction model for hypertension increased the C-statistic by 0.5% for the SBP PRS and 0.6% for the DBP PRS. They also observed that incorporating both PRSs in the clinical prediction model resulted in a 0.7% increase in the C-statistic. Our machine learning models incorporated multiple genetic liabilities in the model and showed a 1% improvement in the classification of hypertension. Our research is unique in that it included ten genetic liabilities (incorporating a total of 883 SNPs) utilizing machine learning to establish a more integrated strategy in the classification of hypertension. Our study also took a different approach in terms of the type of genetic liabilities used. Instead of incorporating hypertension genetic liability, we included genetic liabilities for risk factors associated with hypertension and CVD (see methods). The combination of multiple genetic liabilities implemented within machine learning models for the classification of hypertension is the novelty of our work.

Compared with the study by Niu and colleagues, our study achieved a lower performance. Also, in terms of the net reclassification improvement in prediction value, our study showed only a marginal improvement, whereas the study by Niu and colleagues showed an improvement of up to 4.7% in prediction value. This implies that incorporating genetic liabilities relating to the risk factors of hypertension may not be as promising as incorporating the genetic liability of hypertension itself. However, it should be noted that our study investigated a large-scale European ancestry population and the study by Niu, and colleagues investigated a population of Asian ancestry in rural China (The Henan Rural Cohort Study). These two populations have significant differences in their genetic make-up. Another reason for the observed differences could be environmental exposures and lifestyle variables, which can play a role in modifying the expression or impact of these genetic variants on phenotypes across populations [28,87]. Furthermore, Niu and colleagues did not examine the data-balancing strategy. We employed the data-balancing technique to ensure that our training set was balanced. We also used a feature selection strategy to identify the best features for our machine learning. In addition, we performed model calibration to select the most robust classification model for hypertension.

Our feature selection approach was successful in creating machine learning models that slightly improved the classification of hypertension. However, this came at the price of clinically relevant features (e.g., diabetes mellitus and drinking status) being excluded. We used a specific definition for diabetes (diabetes diagnosed by a doctor, or use of diabetic medication, or Hb1Ac ≥ 48 mmol/mol, or glucose level ≥ 7.0 mmol/dL) [45]. However, the literature shows that diabetes mellitus and hypertension may co-exist, and it is not exactly clear which of the two precedes the other [88,89]. The observation that our machine learning feature selection approach did not prioritize diabetes as an important feature in classifying hypertension may align with the existing inquiries in the literature regarding the extent to which diabetes influences the development of hypertension or conversely [90].

Our models included lifestyle-related factors, such as BMI, smoking, sedentary lifestyle, age, TC, LDL cholesterol, and HDL cholesterol, as well as genetic liabilities for TC and LDL, which were identified as important features in our best classification model for hypertension. Evidence from the existing literature shows that obesity is accompanied by an increased risk of hypertension due to modifying other risk factors (e.g., elevated levels of LDL cholesterol, reduced levels of HDL cholesterol, and elevated blood pressure) in obese individuals [91]. In addition, high cholesterol levels have been linked to an increased risk of developing hypertension [83]. Furthermore, it has also been shown in previous studies that genetic factors in combination with environmental factors may increase the risk of hypertension and other CVDs [44,92,93]. For example, we have recently shown that physical inactivity in combination with high genetic susceptibility to obesity could increase the risk of hypertension [94]. Our current study did not test for interactions between genetic and lifestyle factors; however, both factors were identified as being important in developing the risk of hypertension.

In our study, we employed random forest and neural network methods, which can effectively capture the hidden interactions between genetic and non-genetic factors in hypertension by leveraging their ability to model nonlinear relationships, handle high-dimensional data, and automatically learn relevant features from the data [62]. These techniques provide excellent tools for examining the complicated aetiology of hypertension and identifying important factors that contribute to its development and progression.

### 4.1. Strength

A strength of our study is in the novelty of the approaches used including (1) the use of machine learning to build a prediction model of hypertension in a European setting, (2) testing various methods of feature selection to identify the best performing set of predictive features and to ensure that the features included in the final model were robust and that the model was well calibrated, and (3) the addition of multiple genetic liabilities in one single prediction model to identify the best performing classification model. In our integrated genetic approach, we included multiple genetic liabilities comprising a large number of SNPs within ten genetic liabilities and allowed machine learning to identify the best pattern of feature combination in terms of model performance and accuracy. This gave us a comprehensive picture of the effectiveness of various genetic liabilities in comparison with each other and hypertension risk factors. Another strength is in the use of the large sample size of the UKB that allowed us to develop a large training set comprising 171,304 participants. This is beneficial in detecting the true effect of risk factors on outcomes, reducing bias, and making risk predictions in the testing set more reliable [95,96]. Our study contributes to the ongoing research on the potential role of genetic liabilities in risk prediction of complex diseases [97,98,99].

### 4.2. Limitations

A limitation of our research is that the UKB data are imbalanced in terms of the ratio of cases and controls, and, as a result, our sample included 10,528 more controls than cases. The training set included 7370 more controls than cases. Models trained on imbalanced datasets may become biased towards the dominant class, predicting the minority class incorrectly [58]. To address the imbalance in the dataset and minimize error, we utilized an over-sampling approach to balance the sample [100]. The “*ROSE*” package that we used for balancing our data uses an over-sampling approach that may introduce noise into the synthetic sample in the dataset, resulting in some level of bias remaining in the models [101]. In addition, in this study, we employed an integrative approach incorporating multiple features into a machine learning model. To minimize overfitting due to including potentially irrelevant features, we used 5-fold cross-validation techniques using “*caret*” package which allowed us to evaluate the model’s performance across multiple training set subsets. However, despite the use of cross-validation techniques, there could still be some residual overfitting in the data due to the model’s potential complexity [102]. To mitigate and address the issue of potentially irrelevant features causing overfitting and to improve robustness and generalization as well as performance of our machine learning models, we adopted random forest and neural network, as feature selection techniques, to concentrate on the most significant features to build our machine learning models in stage two.

## 5. Conclusions

Our research highlighted that out of the ten genetic liabilities examined in our study, genetic liability for two lipid traits (TC and LDL) was found to improve the classification of hypertension within a European population. Incorporating these two genetic liabilities in the random forest model slightly improved hypertension risk discrimination and risk reclassification for participants beyond conventional risk factors.

To improve the generalizability and robustness of classifying hypertension, we propose that future studies incorporate multiple genetic liabilities in machine learning-based models.

## Figures and Tables

**Table 1:** Published GWAS SNPs for calculating the genetic liabilities.

Trait Category	Genetic Liability	Study (Publication Year)	Number of SNPs	Reference
Smoking	Smoking initiation	Liu et al., 2019 [49]	311	Liu et al., 2019 [49]
	Smoking cessation	Liu et al., 2019 [49]	16	Liu et al., 2019 [49]
	Smoking heaviness	Liu et al., 2019 [49]	38	Liu et al., 2019 [49]
Diabetes	Type 2 diabetes	Mahajan et al., 2018 [46]	210	Mahajan et al., 2018 [46]
Adiposity	BMI	Winkler et al., 2016 [47]	159	Winkler et al., 2016 [47]
	WHR	Shungin et al., 2015 [48]	39	Shungin et al., 2015 [48]
Lipid traits	TC	Surakka et al., 2015 [50]	36	Surakka et al., 2015 [50]
	HDL	Surakka et al., 2015 [50]	19	Surakka et al., 2015 [50]
	LDL	Surakka et al., 2015 [50]	30	Surakka et al., 2015 [50]
	Triglycerides	Surakka et al., 2015 [50]	25	Surakka et al., 2015 [50]

GWAS: genome-wide association studies, SNPs: single nucleotide polymorphisms, BMI: body mass index, WHR: waist–hip-ratio, TC: total cholesterol, HDL: high-density lipoprotein, LDL: low-density lipoprotein.

**Table 2:** Features included in the machine learning algorithm.

Feature Category	Feature Type	Feature
Characteristics features		Sex Age
Lifestyle-related features	Phenotype	Smoking status Sedentary lifestyle Drinking status
	Genetic	Genetic liability for smoking heaviness Genetic liability for smoking cessation Genetic liability for smoking initiation
Diabetes-related features	Phenotype	Diabetes (see methods for definition)
	Genetic	Genetic liability for type 2 diabetes
Adiposity-related features	Phenotype	BMI
	Genetic	Genetic liability for BMI Genetic liability for WHR
Lipid-related features	Phenotype	TCL DLH DL
	Genetics	Genetic liability for LDL Genetic liability for HDL Genetic liability for TC Genetic liability for triglycerides

BMI: body mass index, WHR: waist–hip ratio, TC: total cholesterol, HDL: high-density lipoprotein, LDL: low-density lipoprotein.

**Table 3:** Baseline characteristic of the UKB participants within the overall sample and hypertensive subgroups.

	Hypertensive	Non-Hypertensive	Overall	*p*-Value
	N = 117,095	N = 127,623	N = 244,718	
Diabetes diagnosed by a doctor:				
YES; N (%)	4697 (4.00%)	2314 (1.80%)	7011 (2.9%)	<0.001
NO; N (%)	112,398 (96.0%)	125,309 (98.2%)	237,707 (97.1%)	
Age (years); mean (SD)	57.6 (7.53)	53.4 (7.84)	55.4 (7.98)	<0.001
BMI (kg/m^2^); mean (SD)	28.0 (4.83)	25.8 (4.06)	26.8 (4.58)	<0.001
TC (mmol/L); mean (SD)	6.05 (1.06)	5.79 (1.04)	5.91 (1.06)	<0.001
HDL (mmol/L); mean (SD)	1.46 (0.38)	1.51 (0.38)	1.49 (0.38)	<0.001
LDL (mmol/L); mean (SD)	3.84 (0.81)	3.62 (0.80)	3.73 (0.81)	<0.001
Sedentary lifestyle (h/day); mean (SD)	4.87 (2.39)	4.50 (2.33)	4.68 (2.37)	<0.001
Sex:				
Male; N (%)	55,686 (47.6%)	47,101 (36.9%)	10,2787 (42.0%)	<0.001
Female; N (%)	61,409 (52.4%)	80,522 (63.1%)	141,931 (58.0%)	
Drinking status:				
Current; N (%)	109,655 (93.6%)	119,884 (93.9%)	229,539 (93.8%)	<0.001
Never; N (%)	4052 (3.46%)	3967 (3.11%)	8019 (3.3%)	
Previous; N (%)	3388 (2.89%)	3772 (2.96%)	7160 (2.9%)	
Smoking status:				
Current; N (%)	37,458 (32.0%)	39,476 (30.9%)	76,934 (31.4%)	<0.001
Never; N (%)	78,292 (66.9%)	86,555 (67.8%)	164,847 (67.4%)	
Previous; N (%)	1345 (1.15%)	1592 (1.25%)	2937 (1.2%)	

Table is generated with gtsummary package using Pearson’s chi-squared test and Wilcoxon rank sum test. SD: standard deviation, BMI: body mass index, TC: total cholesterol, HDL: high-density lipoprotein, LDL: low-density lipoprotein.

**Table 4:** Discrimination and calibration results of the models applied to the testing set.

Classification Models	Numb of Features	R^2^	AUC% (95% Cl)	Brier Score	Spiegel Halter z Score	Spiegel Halter *p*-Value	Slope	Intercept	Accuracy % (95% Cl)	Sensitivity (Recall)	F1 Score
Models with conventional risk factors											
Random forest	10	0.17	0.70 (0.70, 0.71)	0.22	1.03	0.30 *	0.98	0.04	0.65 (0.64, 0.65)	0.68	0.64
Neural network	10	0.19	0.72 (0.71, 0.7)	0.21	−14.39	6.4 × 10^−47^	1.18	0.08	0.66 (0.65, 0.66)	0.69	0.66
Models with conventional risk factors and genetic liabilities											
Random forest	20	0.18	0.71 (0.71, 0.72)	0.22	−5.64	1.7 × 10^−8^	1.06	−0.04	0.65 (0.64, 0.65)	0.68	0.65
Neural network	20	0.19	0.72 (0.71, 0.72)	0.21	−14.44	3.0 × 10^−47^	1.18	0.07	0.66 (0.65, 0.66)	0.68	0.66
Random forest as feature selection method											
Random forest	10	0.17	0.71 (70, 0.71)	0.22	0.10	0.92	0.99	−0.04	0.65 (0.64, 0.65)	0.66	0.64
Neural network	10	0.18	0.72 (0.71, 0.72)	0.21	−15.51	3.1 × 10^−54^	1.20	−0.09	0.66 (0.65, 0.66)	0.69	0.66
Neural network as feature selection method											
Random forest	10	0.16	0.70 (0.70, 0.71)	0.22	−0.44	0.66	1.00	−0.04	0.64 (0.64, 0.65)	0.66	0.64
Neural network	10	0.17	0.71 (0.70, 0.71)	0.22	−13.80	1.6 × 10^−43^	1.18	−0.08	0.65 (0.65, 0.66)	0.69	0.65

* *p*-value > 0.05 (test is not significant) good calibration. Model with conventional risk factors included age, sex, BMI, diabetes, smoking status, drinking status, TC, HDL, LDL, and sedentary lifestyle. Discrimination is measured by the AUC. Accuracy is the percentage of true predictions made by our model out of all predictions made; the F1 is a single score that balances both precision and recall (sensitivity). Recall is the proportion of true positive predictions among all positive instances in the dataset. The Brier score is a combined measure of discrimination and calibration. Calibration is measured by the Spiegelhalter z test, logistic slope, and intercept. BMI: body mass index, AUC: area under the curve, TC: total cholesterol, HDL: high-density lipoprotein, LDL: low-density lipoprotein.

**Table 5:** Net reclassification and integrated discrimination index.

Feature Selection Method	Classification Method	NRI^>0^ (95% Cl) *p*-Value	IDI (95% Cl) *p*-Value
None	* Random forest	Ref	Ref
Random forest	Random forest	0.06 (0.05, 0.08) *p*-value < 0.00001	1.7 × 10^−3^ (9.0 × 10^−4^, 2.5 × 10^−3^) *p*-value = 1.0 × 10^−5^
Neural network	Random forest	−0.10 (−0.12, −0.09) *p*-value < 0.00001	−0.01 (−9.3 × 10^−4^, −0.01) *p*-value < 0.00001

* The random forest model with all conventional risk factors was selected as a reference model. NRI^>0^: continuous net reclassification index; IDI: integrated discrimination index, Cl: confidence interval, Ref: reference.

## Data Availability

Data are contained within the article and supplementary materials.

## Supplementary Materials

The following supporting information can be downloaded at https://www.mdpi.com/article/10.3390/jcm13102955/s1, Supplementary Data S1–Supplementary Data S10: List of genetic variants’ summary statistics used to construct the genetic risk scores, Supplementary Table S1–Supplementary Table S4, Supplementary Figure S1–Supplementary Figure S7.
