# Supplementary material for: Using Machine Learning to Evaluate the Value of Genetic Liabilities in the Classification of Hypertension within the UK Biobank
Source: J Clin Med. 2024 May 17;13(10):2955. doi: 10.3390/jcm13102955 (PMC11122671; doi:10.3390/jcm13102955)
Supplement: Supplementary file 1 [file jcm-13-02955-s001.zip › Maccarthy et. al supplementary_1 (2).pdf]

Supplementary Information for

Using Machine Learning to Evaluate the Value of Genetic Liabilities in the Classification of Hypertension within the UK Biobank

Gideon MacCarthy and Raha Pazoki

Supplementary Table S1: Optimal metrics identified in the random forest models for the classification of hypertension based on the training data.

Model	Number of Features	technique/ set	Outcome	Mtry	OOB Prediction error (Brier s.)
**Stage one**					
Random forest	10	Random forest /training set	Hypertension	3	0.22
Random forest	20	Random forest /training set	Hypertension	4	0.22
**Stage two**					
Random forest†	10	Random forest /training set	Hypertension	3	0.22
Random forest††	10	Random forest /training set	Hypertension	3	0.22

†Model built with top ten important features selected by random forest that include conventional risk factors and genetic liabilities. ††Model built with top ten important features selected by neural network that include conventional risk factors and genetic liabilities. Results are from the *ranger* function within the *ranger* R package. Mtry: the number of variables to randomly sample as candidates at each split, OOB: out of bag.

Supplementary Table S2: Optimal metrics identified in the neural network models for the classification of hypertension based on the training data.

Model	Number of Features	Number of Hidden layers	ROC (SD)	Sensitivity (SD)	Specificity (SD)
**Stage one**					
Neural network	10	5	0.70 (0.004)	0.62 (0.006)	0.67 (0.004)
Neural network	20	5	0.70 (0.004)	0.62 (0.006)	0.67 (0.006)
**Stage two**					
Neural network †	10	5	0.70(0.004)	0.62(0.007)	0.67(0.006)
Neural network ††	10	5	0.69(0.004)	0.61(0.007)	0.66(0.007)

†Model built with top ten important features selected by random forest that included conventional risk factors and genetic liabilities. ††Model built with top ten important features selected by neural network that included conventional risk factors and genetic liabilities. Results are from the *nnet* function within the *caret* R package. The neural network model was built with parameter for weight decay (decay =0), maximum number of iterations (maxit=100), The maximum allowable number of weights (MaxNWts=1000). ROC: receiver operating characteristic curve, SD: Standard deviation.

Supplementary Table S3: Overview of the association analysis between study predictors and hypertension based on univariable logistic regression analysis.

Characteristic	N	OR	95% CI	*p-value*
Diabetes Mellitus (yes)	244,718	2.26	2.15, 2.38	<0.001
Sex (Male)	244,718	1.55	1.53, 1.58	<0.001
Age	244,718	1.07	1.07, 1.07	<0.001
BMI	244,718	1.12	1.12, 1.12	<0.001
Smoking Status	244,718			
Current	reference	1.0	—	
Never		1.15	1.12, 1.18	<0.001
Previous		1.32	1.28, 1.36	<0.001
Drinking Status	244,718			
Current	reference	1.0	—	
Never		1.12	1.07, 1.17	<0.001
Previous		0.98	0.94, 1.03	0.4
Total Cholesterol	244,718	1.27	1.26, 1.28	<0.001
HDL	244,718	0.72	0.70, 0.73	<0.001
LDL	244,718	1.39	1.37, 1.40	<0.001
Sedentary Lifestyle	244,718	1.07	1.07, 1.07	<0.001

OR: Odds Ratio, CI: Confidence Interval

Supplementary Table S4: Overview of the association analysis between study predictors and hypertension based on multivariable logistic regression analysis.

Characteristic	N	OR	95% CI	*p-value*
Diabetes Mellitus(yes)	244,718	1.56	1.48, 1.65	<0.001
Sex(male)	244,718	1.70	1.67, 1.73	<0.001
Age	244,718	1.07	1.07, 1.08	<0.001
BMI	244,718	1.12	1.12, 1.13	<0.001
Smoking Status	244,718			
Current	reference	1.0	—	
Never		1.11	1.08, 1.14	<0.001
Previous		1.07	1.04, 1.10	<0.001
Drinking Status	244,718			
Current	reference	1.0	—	
Never		0.98	0.94, 1.03	0.5
Previous		0.93	0.88, 0.97	0.003
Total Cholesterol	244,718	1.66	1.59, 1.73	<0.001
HDL	244,718	0.91	0.87, 0.94	<0.001
LDL	244,718	0.63	0.60, 0.66	<0.001
Sedentary Lifestyle	244,718	1.01	1.01, 1.01	<0.001

OR: Odds Ratio, CI: Confidence Interval

Supplementary Figure S1:

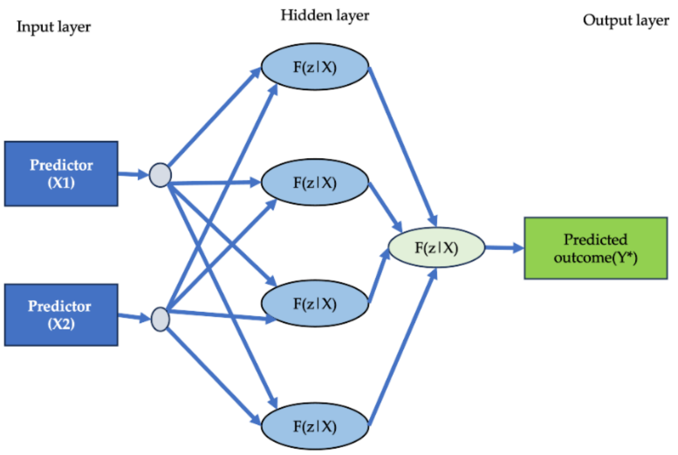

Supplementary Figure S1: A schematic architecture of neural network.
F(z|x): Black box function

Supplementary Figure S2

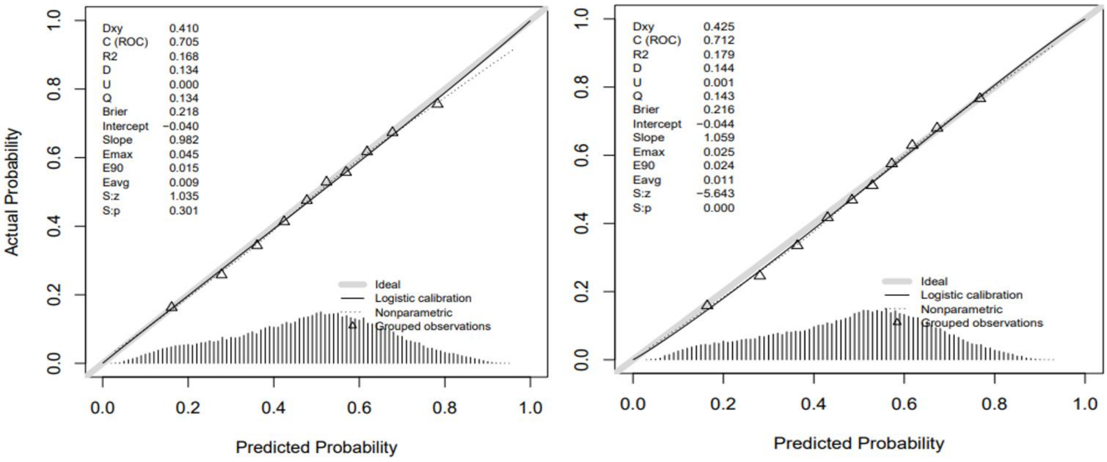

Supplementary Figure S2: Calibration curve of stage one random forest models. The random forest with conventional risk factors (left panel) is well-calibrated. The random forest model that included conventional risk factors and genetic liabilities (right panel) is poorly calibrated due to overfitting. The solid grey line is ideal calibration, the solid black line is logistic calibration, the dotted line is a non-parametric calibration, and the triangular points are the grouped observations. The distribution plot of predicted probability is also displayed.

Supplementary Figure S3

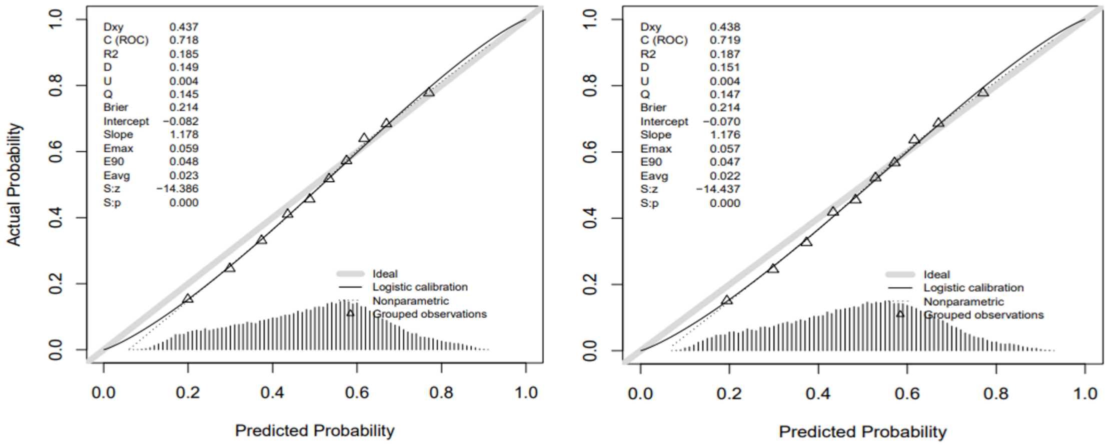

Supplementary Figure S3: Calibration curve of stage one neural network models. The neural network with conventional risk factors (left panel) as well as the neural network model that included conventional risk factors and genetic liabilities (right panel) are both poorly calibrated due to overfitting. The solid grey line is ideal calibration, the solid black line is logistic calibration, the dotted line is a non-parametric calibration, and the triangular points are the grouped observations. The distribution plot of predicted probability is also displayed.

Supplementary Figure S4

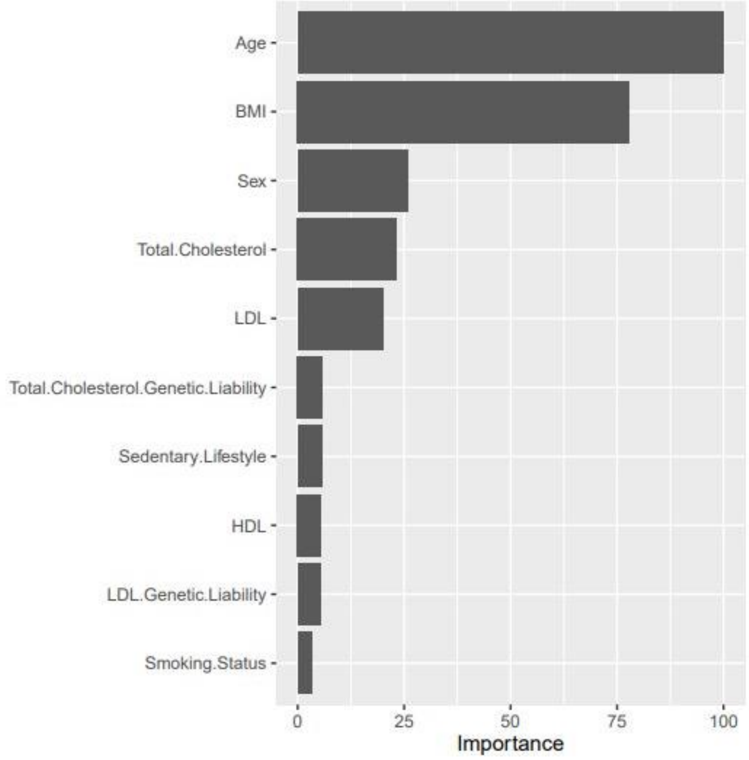

Supplementary Figure S4: Top ten important features selected by the random forest model that include genetic liabilities in addition to conventional risk factors.

Supplementary Figure S5

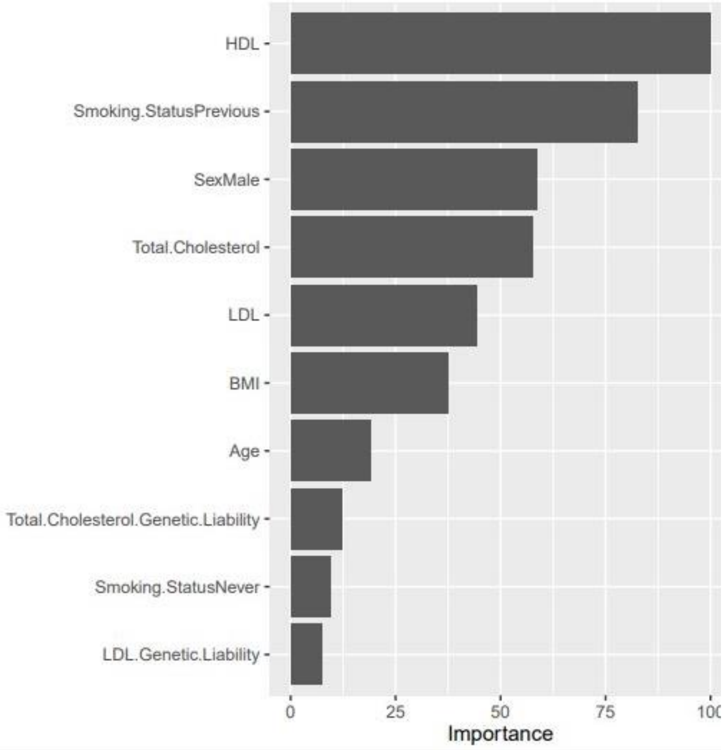

Supplementary Figure S5: Top ten important features selected by the neural network model that include genetic liabilities in addition to conventional risk factors.

Supplementary Figure S6

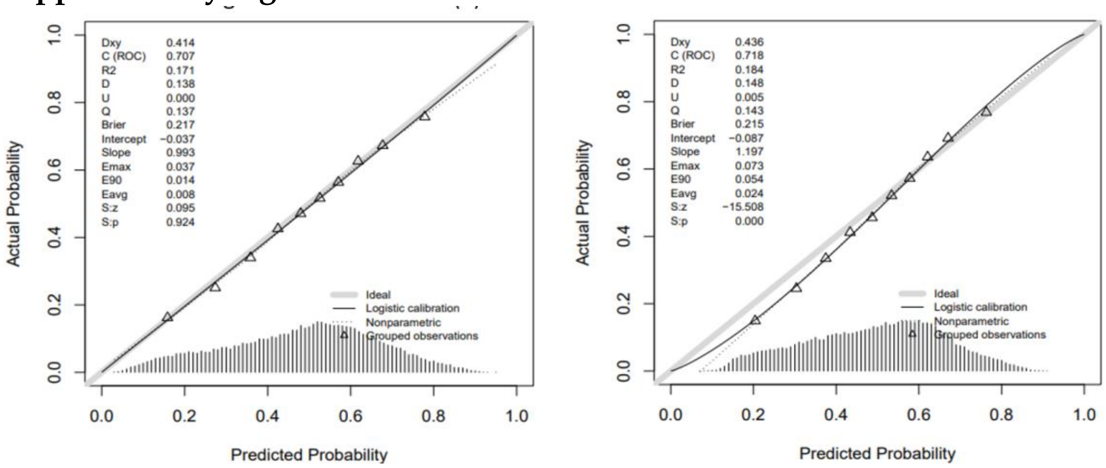

Supplementary Figure S6: Calibration curve of stage two models created with features selected by random forest model. The model classified with random forest (left panel) is well-calibrated. The model classified with neural network (right panel) is poorly calibrated due to overfitting. The solid grey line is ideal calibration, the solid black line is logistic calibration, the dotted line is a non-parametric calibration, and the triangular points are the grouped observations presence. The distribution plot of predicted probability is also displayed.

Supplementary Figure S7

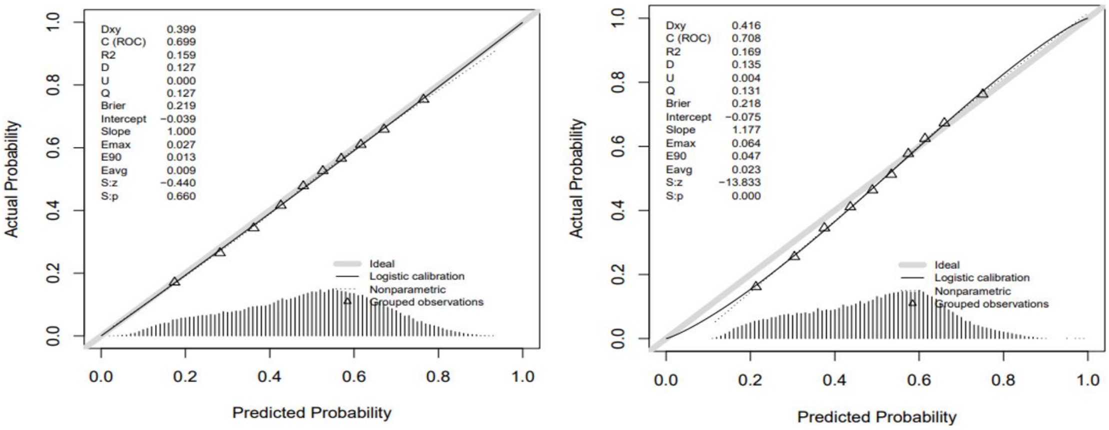

Supplementary Figure S7: Calibration curve of stage two models created with features selected by neural network model. The model classified with random forest (left panel) is well-calibrated and neural network (right panel) is poorly calibrated due to overfitting. The solid grey line is ideal calibration, the solid black line is logistic calibration, the dotted line is a non-parametric calibration, and the triangular points are the grouped observations presence. The distribution plot of predicted probability is also displayed.
